# Voxelotor: A Ray of Hope for Sickle Disease

**DOI:** 10.7759/cureus.7105

**Published:** 2020-02-26

**Authors:** Salma M AlDallal

**Affiliations:** 1 Hematology Laboratory Specialist, Amiri Hospital, Kuwait City, KWT

**Keywords:** sickle cell disease, voxelotor, us fda

## Abstract

Sickle cell disease is one challenging blood disorder, affecting around 100,000 people in the United States alone. None of the currently approved drugs can modify the underlying pathology of the disease. Voxelotor, first of its kind, is an orally administered drug that can alter the underlying disease pathology (by increasing the affinity between Hb and oxygen) and inhibit sickling of red blood cells. Several clinical trials and case series have documented the benefits and safety of voxelotor therapy in sickle cell disease.

Currently, the US FDA has approved the drug for treatment of sickle cell disease and also granted the status of orphan drug.

## Introduction and background

Sickle cell disease is a group of autosomal recessive diseases occurring following mutation of the β chain of hemoglobin (substitution of a single amino acid in the β chain of hemoglobin), and resulting in the production of abnormal sickle hemoglobin (HbS) [1]. There are different types of sickle cell disease; the most common are sickle cell anemia, sickle hemoglobin-C disease, sickle beta-plus thalassemia, and sickle beta-zero thalassemia. Deoxygenation of hemoglobin results in the polymerization of HbS and production of deformed red blood cells (RBCs; sickle shaped). Sickling of the RBCs ultimately leads to permanent damage to the RBC cell membrane [1-3]. All these abnormal RBCs lead to hemolysis, chronic anemia, increased risk of inflammation and finally vaso-occlusion. Sickle cell disease can lead to array of signs and symptoms namely sickle cell crisis, vaso-occlusive crisis (leading to pain, ischemia, necrosis, and finally organ damage), splenic sequestration crisis, acute chest syndrome, aplastic crisis, hemolytic crisis, and others (like dactylitis, one of the earliest manifestations). Chronic anemia and hemolysis lead to tissue hypoxia and multiple organ damage and, therefore, increase the risk of untimely demise [1-5]. Around 100,000 people are affected by sickle cell disease in the United States alone, and the disease considerably reduces life expectancy by approximately 30 years [1].

## Review

Available drugs for sickle cell disease

Approved drug therapy for sickle cell disease includes L-glutamine and hydroxyurea [6-8]. To date, there are no approved drugs that can modify the underlying disease mechanism of sickle cell disease. Among the two approved drugs, hydroxyurea is helpful only in some patients of sickle cell disease and has raised issues regarding its safety like a significant degree of myelosuppression and teratogenicity [7]. L-Glutamine, a recently approved drug for sickle cell disease, does not target the underlying disease pathology in sickle cell disease patients but rather produces its modest effect by preventing the vaso-occlusive crisis period to some extent without improving the hematological parameters [8]. Thus, there is a need for a drug that can modify the underlying pathology of the disease and, therefore, prevent the long-term complications of the disease.

It is understood that the oxygenation of HbS inhibits the polymerization of HbS; hence, by increasing the level of oxygenated HbS, the underlying pathology of sickle cell disease can be modified [1-5,6]. Earlier other Hb allosteric modifiers like 5‐hydroxymethylfurfural (5‐HMF), valerosol (BW12C), and tucaresol were explored for their potential roles in sickle cell disease [1,5,6]. Tucaresol and valerosol have shown to decrease the sickling of RBCs (in vitro) and the incidence of hemolysis in sickle cell disease without reducing oxygen supply to different tissues by increasing the affinity between Hb and oxygen by 20%-30% [1,6]. However, poor pharmaceutical characteristics of 5-HMF and lack of specific action and highly immunogenic properties of tucaresol led to no further investigation of these drugs [1-6].

Voxelotor

Voxelotor (earlier known as GBT440) is the first of its kind oral medication for the treatment of sickle cell disease. This drug acts by modifying the affinity between Hb and oxygen. It acts through a covalent bond (reversible in nature) formation with the amino acid valine located at the N terminal of the α chain of Hb. It results in the allosteric modification of Hb and increases the affinity between oxygen and Hb [5,6].

As it has been demonstrated that the oxygenation of HbS prevents its sickling, there is a high probability that voxelotor might inhibit the sickling of HbS and, thus, modify the disease outcome by interfering with the underlying pathology.

Preclinical study of voxelotor revealed that following exposure of purified HbS with voxelotor, the voxelotor-modified HbS was found to be as useful as fetal Hb as it also delays the polymerization of HbS [9]. In vitro studies carried out with blood samples of sickle cell disease patients and in vivo studies carried out in animals revealed that voxelotor is highly specific for Hb and possesses a favorable half-life requiring once-daily dosing. Furthermore, studies have also shown that voxelotor increases the affinity between Hb and oxygen, decreases sickling of HbS, corrects the deformity of the sickled RBCs, decreases blood viscosity, extends the half-life of RBCs, and exhibits a favorable linear pharmacokinetic vs. pharmacodynamics relationship [9-11].

Voxelotor (brand name Oxbryta) is developed by the Global Blood Therapeutics. The US Food and Drug Administration (FDA) has approved this drug for use in sickle cell disease, and the drug has been given the status of orphan drug in rare pediatric diseases [1,5,6]. The US FDA has also put the trial of this on fast track and designated the drug as breakthrough therapy.

Furthermore, the European Medicines Agency has included voxelotor in the PRIME (PRIority MEdicines) scheme, which provides support for the development of medicines that target still unmet medical needs. The European Commission has also granted orphan drug status to voxelotor for the treatment of sickle cell disease [1,5,6].

Clinical trials on voxelotor

Howard and his colleagues published a phase 1/2 randomized, double-blind, placebo-controlled, single and multiple ascending dose study of voxelotor [12]. The study included both healthy volunteers and sickle cell disease patients. The initial study was followed by an open-label, single-arm extension study for six months. The study evaluated the pharmacokinetic and pharmacodynamic characteristics, safety, and tolerability of voxelotor and established the proof of concept by improving the clinical measures of anemia, episodes of hemolysis, and percentage of deformed RBCs (sickled).

A total of 38 patients of sickle cell disease received voxelotor once daily for 28 days at a dose of 500, 700, or 1,000 mg or placebo. In the second phase, 16 patients received 700 or 900 mg of voxelotor or placebo for 90 days. Four patients from the 90-day cohort were subsequently enrolled in an extension study and treated with 900 mg of oral voxelotor once daily for the next six months. All participants who received voxelotor at different doses for more than 28 days showed significant hematological improvements in terms of increased Hb level, reduction in hemolysis, and the percentage of sickled RBCs, suggesting the role of voxelotor as a disease-modifying agent in sickle cell disease.

The drug was well tolerated in all the participants (28-day cohort, 90-day cohort, and in the subsequent open-label extension study). Both the trials (28-day trial and 90-day trial) were registered separately at www.clinicaltrials.gov (NCT02285088 and NCT03041909, respectively) [12]. Another case report documented that voxelotor treatment led to remission of jaundice in a patient who completed both phase 1 and phase 2 extension trial successfully and improved his quality of life significantly [13].

Moreover, another case series reported by Blyden and his colleagues documented the compassionate use of voxelotor in seven patients suffering from severe sickle cell disease [14]. The treatment options for severe sickle cell disease are rather limited mainly because of the lack of US FDA approved drugs and therapies and also because of systematic exclusion of such patients from ongoing trials for investigational drugs. The seven patients were planned to receive 900 mg of voxelotor once daily, which could be increased up to 1,500 mg. These patients were aged between 22 and 67 years and received treatment between 6 and 17 months. No voxelotor-related adverse events were reported in any of the patients, except for diarrhea in two patients with high dose (1,500 mg/day). Two patients with prior extensive organ damage due to sickle cell disease died during therapy; both deaths were attributed to the progression of the underlying disease and not related to voxelotor therapy.

The same case series also reported that in those severe sickle cell disease patients (Hb<6 g/dL), voxelotor therapy improved Hb levels and oxygen saturation, and also reduced the need for hospital admission for episodes of vaso-occlusive crisis pain in the first 24 weeks of therapy [14]. A pivotal phase 3 trial known as GBT-HOPE (NCT03036813) was carried with voxelotor as test drug to assess its safety and efficacy compared to placebo in sickle cell disease patients [5]. The results showed that voxelotor significantly increased hemoglobin concentration and also reduced the markers of hemolysis.

In this trial, 274 sickle cell disease patients (aged 12 years or more) were randomly assigned to two groups: one group of patients (n=92) received placebo, while the other group received voxelotor 900 mg (n=92) or 1,500 mg (n=90) once daily. Following 24 weeks of therapy, 51.1% of the patients getting higher dose of voxelotor showed a significant rise in hemoglobin levels compared to the rise in hemoglobin in only 6.5% of the participants in the placebo group. The study further found that the above-mentioned findings were also consistent with HbS polymerization, suggesting that voxelotor might have disease-modifying potential in sickle cell disease.

In the same trial, it was found that the incidence of adverse drug reaction in trial participants was similar for both the placebo and the test drug groups. Adverse events (grade 3) were found to affect 26% of the participants taking 1,500 mg of voxelotor, 23% of the participants taking 900 mg of voxelotor, and 26% of the participants in the placebo group. Furthermore, the investigators found that majority of the adverse events were unrelated to drugs (voxelotor or placebo).

## Conclusions

Based on all these published reports, voxelotor seems promising in sickle cell disease, especially as a disease-modifying agent; however, further studies regarding the effect of voxelotor on vaso-occlusive episodes and pain are warranted. Also, more clinical trials comparing voxelotor with other already approved or soon-to-be approved drugs are required.
